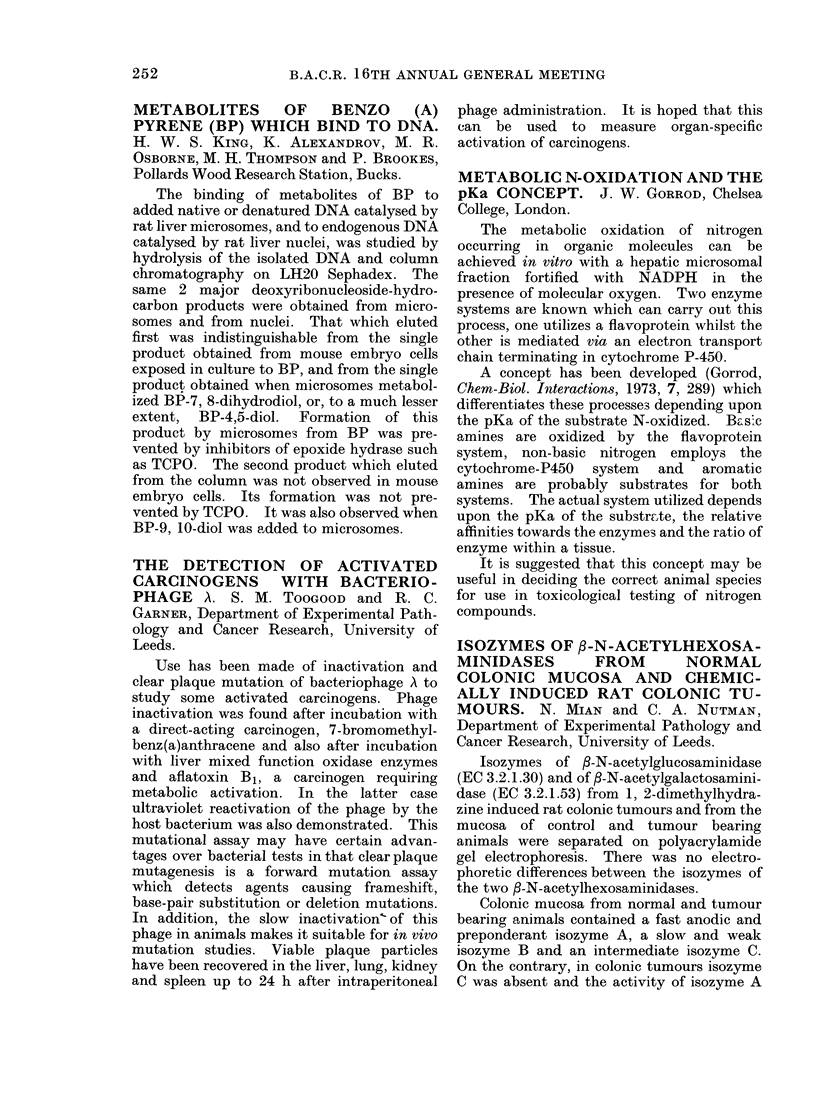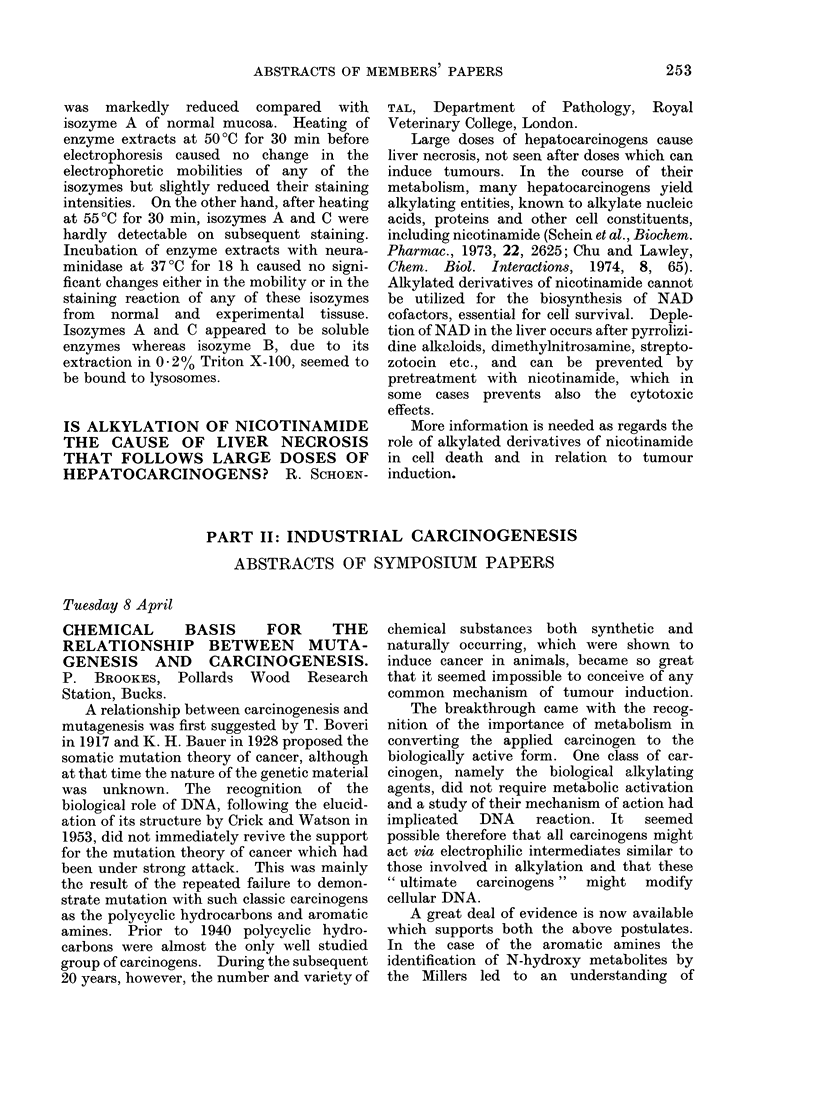# Proceedings: Isozymes of beta-N-acetylhexosaminidases from normal colonic mucosa and chemically induced rat colonic tumours.

**DOI:** 10.1038/bjc.1975.197

**Published:** 1975-08

**Authors:** N. Mian, C. A. Nutman


### ISOZYMES OF β-N-ACETYLHEXOSAMINIDASES FROM NORMAL COLONIC MUCOSA AND CHEMICALLY INDUCED RAT COLONIC TUMOURS.

N. Mian and C. A. Nutman, Department of Experimental Pathology and Cancer Research, University of Leeds.

Isozymes of β-N-acetylglucosaminidase (EC 3.2.1.30) and of β-N-acetylgalactosaminidase (EC 3.2.1.53) from 1, 2-dimethylhydrazine induced rat colonic tumours and from the mucosa of control and tumour bearing animals were separated on polyacrylamide gel electrophoresis. There was no electrophoretic differences between the isozymes of the two β-N-acetylhexosaminidases.

Colonic mucosa from normal and tumour bearing animals contained a fast anodic and preponderant isozyme A, a slow and weak isozyme B and an intermediate isozyme C. On the contrary, in colonic tumours isozyme C was absent and the activity of isozyme A

was markedly reduced compared with isozyme A of normal mucosa. Heating of enzyme extracts at 50 °C for 30 min before electrophoresis caused no change in the electrophoretic mobilities of any of the isozymes but slightly reduced their staining intensities. On the other hand, after heating at 55 °C for 30 min, isozymes A and C were hardly detectable on subsequent staining. Incubation of enzyme extracts with neuraminidase at 37 °C for 18 h caused no significant changes either in the mobility or in the staining reaction of any of these isozymes from normal and experimental tissuse. Isozymes A and C appeared to be soluble enzymes whereas isozyme B, due to its extraction in 0·2% Triton X-100, seemed to be bound to lysosomes.